# Application of Eye Tracking in Puzzle Games for Adjunct Cognitive Markers: Pilot Observational Study in Older Adults

**DOI:** 10.2196/24151

**Published:** 2021-03-22

**Authors:** Christine Krebs, Michael Falkner, Joel Niklaus, Luca Persello, Stefan Klöppel, Tobias Nef, Prabitha Urwyler

**Affiliations:** 1 University Hospital of Old Age Psychiatry and Psychotherapy University of Bern Bern Switzerland; 2 Gerontechnology & Rehabilitation group University of Bern Bern Switzerland; 3 ARTORG Center for Biomedical Engineering Research University of Bern Bern Switzerland; 4 Department of Neurology University Neurorehabilitation unit Inselspital Bern Switzerland

**Keywords:** eye tracking, puzzle games, aging, cognitive assessment, cognition, attention, executive functions, visual search, fixations

## Abstract

**Background:**

Recent studies suggest that computerized puzzle games are enjoyable, easy to play, and engage attentional, visuospatial, and executive functions. They may help mediate impairments seen in cognitive decline in addition to being an assessment tool. Eye tracking provides a quantitative and qualitative analysis of gaze, which is highly useful in understanding visual search behavior.

**Objective:**

The goal of the research was to test the feasibility of eye tracking during a puzzle game and develop adjunct markers for cognitive performance using eye-tracking metrics.

**Methods:**

A desktop version of the Match-3 puzzle game with 15 difficulty levels was developed using Unity 3D (Unity Technologies). The goal of the Match-3 puzzle was to find configurations (target patterns) that could be turned into a row of 3 identical game objects (tiles) by swapping 2 adjacent tiles. Difficulty levels were created by manipulating the puzzle board size (all combinations of width and height from 4 to 8) and the number of unique tiles on the puzzle board (from 4 to 8). Each level consisted of 4 boards (ie, target patterns to match) with one target pattern each. In this study, the desktop version was presented on a laptop computer setup with eye tracking. Healthy older subjects were recruited to play a full set of 15 puzzle levels. A paper-pencil–based assessment battery was administered prior to the Match-3 game. The gaze behavior of all participants was recorded during the game. Correlation analyses were performed on eye-tracking data correcting for age to examine if gaze behavior pertains to target patterns and distractor patterns and changes with puzzle board size (set size). Additionally, correlations between cognitive performance and eye movement metrics were calculated.

**Results:**

A total of 13 healthy older subjects (mean age 70.67 [SD 4.75] years; range 63 to 80 years) participated in this study. In total, 3 training and 12 test levels were played by the participants. Eye tracking recorded 672 fixations in total, 525 fixations on distractor patterns and 99 fixations on target patterns. Significant correlations were found between executive functions (Trail Making Test B) and number of fixations on distractor patterns (*P*=.01) and average fixations (*P*=.005).

**Conclusions:**

Overall, this study shows that eye tracking in puzzle games can act as a supplemental source of data for cognitive performance. The relationship between a paper-pencil test for executive functions and fixations confirms that both are related to the same cognitive processes. Therefore, eye movement metrics might be used as an adjunct marker for cognitive abilities like executive functions. However, further research is needed to evaluate the potential of the various eye movement metrics in combination with puzzle games as visual search and attentional marker.

## Introduction

### Background

From finding certain items among many others (eg, a book in the library) to navigating, visual search is a necessary behavior in our daily life. Visual search is the ability to find and locate target objects in a pool of stimuli [1,2]. To elaborate the large amount of visual information, frequent eye movements termed saccades are performed until the correct target is fixated [3]. Different stimuli compete for attention to get selected for a fixation. Some selective attention models claim that low-level visual features such as intensity, color, and edge orientation increase the probability of gaining visual attention and influencing eye movements such as saccades or fixations. Saccades and fixations are not only related to attention but also to other cognitive functions like memory [2,4,5].

### Visual Search in Aging and Neurodegenerative Diseases

Visual search strategies change in the course of aging and are also affected by age-related neurodegenerative diseases. When compared with younger adults, older adults show decreased peripheral target detection [6]. During memory retrieval, eye movement supports the reinstatement of spatial locations of stimuli and their temporal order during encoding. During aging, gaze reinstatement can support memory performance as compensation when task demands are too high. But this compensation is only possible up to a certain extent after which further compensation is impossible [2]. Older adults have shown worse performance than younger participants in visual search tasks, including longer reaction times and more time spent per item [7]. The absence of targets within a trial as well as an increasing number of distractors have been shown as responsible factors for the decreased performance in older adults [8]. This decreasing efficiency in visual search tasks in older adults can be explained by a decline in visual processes and executive functions [9]. In pathological aging (ie, dementia), saccade abnormalities are correlated with the level of cognitive impairment [10].

### Puzzle Games as a Tool for Investigating Visual Search

**Figure 1:**
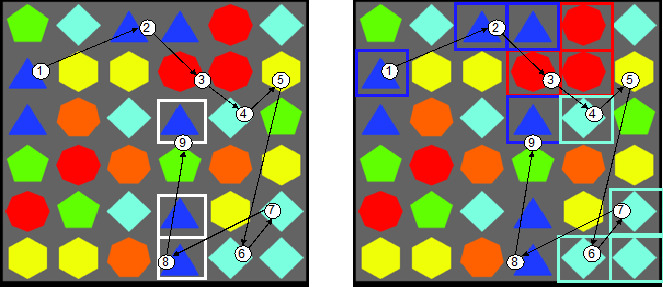
Target pattern (left) and distractor patterns surrounding target pattern (right). Areas of interest for eye tracking are marked, and theoretical gaze path is depicted.

Visual search also plays a key role in solving specific computerized cognitive trainings such as puzzle games [11]. Puzzle games have been used as training tools in health sciences [12-14], and recent research suggests their potential as digital markers of cognitive and motor dysfunctions [15,16]. Well-known puzzle games are tile-matching match-three (TMM3) [17] and flow free [18]. In TMM3 games, participants remove as many tiles as possible in a certain amount of time [17]. Participant must perform visual search to identify where 3 identical puzzle pieces could line up by moving one piece by one place in a grid horizontally or vertically. A typical TMM3 puzzle is ideal for investigating visual search, as the participant has to track the location of multiple static items which requires focused and deliberate searching for target items and visuospatial processing [13,15]. A set of distractor patterns are typically present within each TMM3 puzzle (Figure 1). The filtering of distractor elements has long been established as key in the selection of visual search targets [19]. Observing participant gaze fixations toward distractor patterns may provide additional information on the cognitive processes necessary for solving these tasks [15,20]. In this study, we used a Search and Match Task (SMT) based on a TMM3 video game, a cognitively demanding puzzle game, which has also been demonstrated in other studies [15,21].

### Visual Search in Puzzle Games

Visual search tasks investigate how we find a defined item in a complex environment. In such tasks, participants are typically asked to search for a certain target among distractor items. These target items are defined by one or several distinct features such as shape or color [1]. During visual search three subprocesses are distinguishable: initiation of search, overt search of visual display, and verification of target [3]. The overall process of visual search can be described as either efficient or inefficient; which search type is used depends on the environment. In an efficient search process, the target is easy to identify, and items can be processed in parallel. The inclusion of additional distractors has no effect on the reaction time. In an inefficient search process, target and distractors show similar visual features, and the target is not more salient than the other items. In this case, attention must be allocated to single items in a serial way until the target is found. The addition of distractors increases reaction time [9,22,23]. Certain kinds of puzzle games can be seen as inefficient visual search tasks, where color and shape of items drive attentional search processes [11,24].

During a visual search task, different strategies can be used to allocate attention, depending on the task demands. One way to investigate the type of strategy is assessing the number of eye movements. When participants receive no feedback about task performance, they tend to use similar search strategies across different types of tasks but different amount of saccades can be measured. In an efficient search task, fewer saccades are performed than in an inefficient search task. If feedback about performance is provided, participants are able to adapt strategies to fit the demands of the current task [25]. Eye movements are also influenced by style of stimuli presentation. When stimuli are arranged in grid-like patterns, visual search tasks lead to systematic scanning behavior. There are larger amount of horizontal than vertical saccades during the task, even if the grid-like pattern is heavily distorted (ie, items are displayed irregularly and not on every junction of the grid) [26]. Further research has shown that the stage of verification process takes longer when the grid-like pattern is more distorted while search initiation and scanning time are not affected [3].

Performance in visual search can be increased by the repeated use of puzzle games in the course of a training [21]. Additionally, puzzle games might serve as assessment tools for visual search impairments in patients with neurodegenerative diseases. One study on patients with mild dementia due to Alzheimer disease (AD), for example, reported impairments in shifting attention as well as in the ability to take advantage of visual cues in a visual search task, visible in prolonged reaction times [27].

### Eye Tracking

In visual search tasks, end-of-trial reaction times are commonly assessed but they provide only limited information about how search processes evolved across the trial [20]. One possibility for gathering additional data during visual search tasks is with the use of eye tracking. Eye tracking refers to the process of using an eye-tracker device to track the point of gaze or eye movement of a person. Among other data, it provides gaze coordinates of the user as they search and scan the environment for a certain kind of stimuli [28]. Compared with simple reaction time measurements, eye-tracking data provide insight into visual searching behavior (eg, which item was attended, how long, and when). Therefore, eye-tracking data are useful for understanding fundamental attentional processes (involved in visual search) and strategies [20,28]. Fixations and saccades are commonly analyzed measurements to understand visual attention from eye-tracking data. Fixations are moments when eyes fixate on an object to extract and encode information. The most widely used fixation-based metrics are number of fixations, number of fixations on area of interest, fixation duration, and fixation density. A longer duration of fixation indicates deeper processing of the stimuli and is correlated to high cognitive workload. Saccades are rapid eye movements between fixations. Saccadic metrics include number of saccades, saccadic amplitude (distance), and saccadic duration. Scan paths are a complete description of saccade-fixate-saccade sequences. In video games, eye-tracking data can be used to assess usability and effects of game design [29,30]. Other possible eye-tracking metrics are smooth pursuit, pupil dilation, and blinking. Smooth pursuit movements are slower voluntary movements of tracking dynamic stimuli related to working memory and attention; they have been reported as a diagnostic tool for mild traumatic brain injury [31]. Pupil dilation mirrors cognitive workload and is related to attentional cognitive processes. During the assessment of pupil dilatation, luminance must be controlled because of its effect on pupil diameter, which is stronger than the effect of changes in cognitive workload [32,33]. Blinking has been related to cognitive control [33] and workload [34].

Eye tracking has been used for the assessment of different cognitive processes [35]. It shows potential as a tool to track disease progression, assessing the interplay of both motor and cognitive functions [10]. In a review of eye movements in AD, the authors conclude that patients with AD have different eye movement patterns compared with healthy older adults [36]. The prominent oculomotor features of patients with AD are saccadic intrusions and fixation instability, which are explained by the impairment of saccade pathways in AD. Visual exploration studies have shown that saccades are shorter and fixations are longer in patients with AD than in healthy older adults [37]. Also, higher number of fixations are reported in patients with AD during visual search [38], and the extent of saccade abnormalities in dementia is related to the level of cognitive impairment. To use eye movements as digital markers, they must provide replicable results across the short term and be related to disease severity [10]. To assess the possibility of using eye-tracking data as digital markers for cognitive performance in visual search tasks, it is necessary to study the feasibility of eye tracking in such a task in a first step. This study only addressed saccades and fixations from the eye-tracking measures to study the relationship of eye movements and cognition.

### Research Questions

The goal of this study was to test the feasibility of eye tracking with puzzle games to obtain adjunct markers for cognitive processes using eye-tracking metrics such as fixations and saccades. To this purpose, we used the SMT combined with a stationary eye-tracking device and conducted a preliminary user study in older adults to evaluate the feasibility of the setup and eye-tracking metrics. First, we expected that game completion time is influenced by the number of fixations. Second, we expected a relationship between the fixations and different difficulty levels of the puzzle game. Third, we expected an association between fixations and saccades and assessment scores for cognition and executive function.

## Methods

### Participants

Healthy older subjects (n=13; 5 women; mean age 70.67 [SD 4.75] years; range: 63 to 80 years) were recruited from another ongoing study at the University Hospital of Old Age Psychiatry and Psychotherapy in Bern. Inclusion criteria were ability to consent in study participation, age between 60 and 85 years, native or fluent German speaker, and normal or corrected to normal vision and hearing. Exclusion criteria were any history of seizure or stroke, traumatic brain injury, smoking, psychotropic medication, severe tinnitus, self-reported left-handedness, and cognitive impairment (Montreal Cognitive Assessment [MoCA] score <26) [39-41]. No compensation for participation was provided. All participants provided written informed consent prior to study onset in accordance with the Declaration of Helsinki. The cantonal ethics committees of Bern and Northwest and Central Switzerland granted the ethics approval for this study (2016-01281).

### Neuropsychological Assessment and Game Perception Questionnaires

The study was performed in one session of approximately 40 minutes per participant. The standardized neuropsychological assessments were administered in paper-pencil format prior to the computer-based puzzle task. The neuropsychological tasks were to assess the concurrent criterion validity of their abilities in relation to the puzzle game assessment and keep consistent findings with the study’s previous work [11]. The assessment included the German version of the MoCA [39], Trail Making Test (TMT) A and B [42], Snellgrove Maze Test (SnMT) [43], and Lawton Instrumental Activities of Daily Living (IADL) questionnaire [44]. Cognitive health is defined using the MoCA [41], while the Lawton-IADL is a measure of functional impairment. Additionally, the perception of game [45] and the system usability scale [46] questionnaires were administered in paper-pencil format after completion of the computer-based puzzle-game task.

Demographics and neuropsychological assessment scores of the 13 recruited participants are reported in Table 1. All participants were right-handed except one person (ID 2, ambidexter). Table 1 shows that all participants were cognitively (MoCA: 27.69 [SD 1.374], range 26-30; TMT-A: 20.82 [SD 4.05] sec, range 12.10-28.2 sec; TMT-B: 89.87 [SD 35.47] sec, range: 47.05-128.74 sec; SnMT: 30.84 [SD 10.95], range 15.43-52.26 sec) and functionally (IADL: 7.62 [SD 0.65], range 6-8; cutoff <5) [47] healthy.

**Table 1:** Participant characteristics and demographics.

Subject	Age (years)	Gender	Glasses	MoCA^a^	TMT-A^b^ (sec)	TMT-B^c^ (sec)	SnMT^d^ (sec)	IADL^e^
1	73	m	Yes	26	23.85	173.00	25.53	8
2	72	m	No	27	22.06	108.00	25.86	8
3	66	m	Yes	27	18.18	54.07	—^f^	6
4	80	m	No	26	21.92	85.45	36.12	8
5	71	m	Yes	27	21.80	84.50	52.26	8
6	65	m	Yes	30	17.26	51.15	34.10	8
7	70	f	Yes	29	19.38	118.89	27.48	8
8	68	f	No	27	20.05	85.00	30.95	7
9	76	m	Yes	29	18.51	89.49	17.83	8
10	73	m	Yes	27	21.60	128.74	28.46	7
11	71	f	No	28	25.80	80.00	49.00	8
12	63	f	No	30	12.10	47.05	15.43	7
13	73	f	No	27	28.20	63.00	27.00	8

^a^MoCA: Montreal Cognitive Assessment (max 30).

^b^TMT-A: Trail Making Test A.

^c^TMT-B: Trail Making Test B.

^d^SnMT: Snellgrove Maze Test.

^e^IADL: Lawton Instrumental Activities of Daily Living (max 8).

^f^Not available.

### Puzzle Game: Search and Match Task

**Figure 2:**
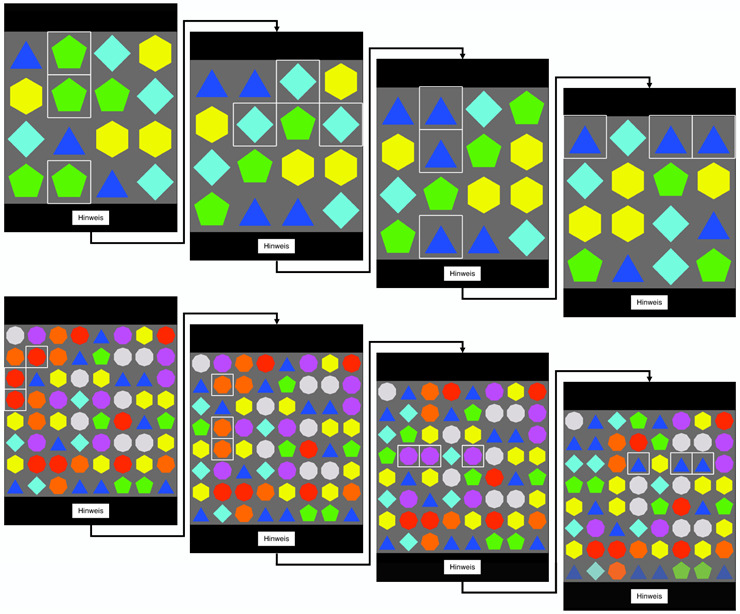
Tile-matching match-three game and step-by-step visual guide for solving each stage of a single 4-step round. In this version of the game, levels were carefully designed so that only one possible solution exists in each step [15].

A custom version of TMM3 SMT game was designed according to the specifications of Chesham et al [15]. The game was built in Unity 3D (language: C#). Modifications to the game were made to include Tobii Pro’s eye-tracking Unity plugin (Unity Technologies) and the recording of data, which also included porting the game to a windows platform from the previous iPad app. This pattern-matching visual search task was played on a grid-based puzzle board filled with colored shapes (ie, tiles). The goal of the task was to produce a vertical or horizontal line with 3 identical tiles. This was reached by swapping 2 neighboring tiles (Figure 2). Only moves that produced a target sequence were allowed or else the tiles bounced back to their original place. Level of difficulty was associated with set size and number of unique tiles/gems as defined by Chesham et al [11]. At the beginning of the puzzle game task, 3 training trials were presented. This was followed by 12 difficulty levels selected from a predefined subset that were randomized without any predetermined progression. Each difficulty level was designed to facilitate 4 boards. Each board contained a single-target visual search task (match-3 solution) and was self-terminating (ended as soon as single target pattern was made). Game-based search time, number of hints used, and number of false moves [11] were collected for the 3 training and 12 test levels. Distractor patterns were not controlled but recorded for each level played.

### Experiment and Data Collection

**Figure 3:**
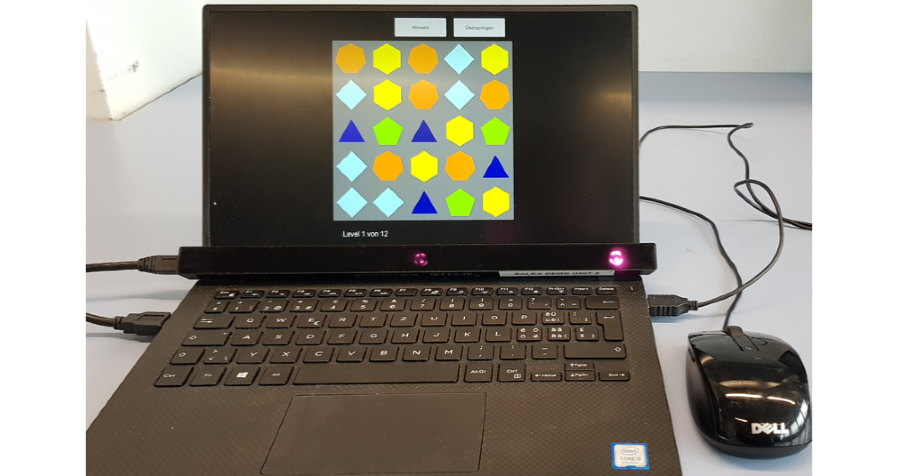
Setup presented to participants during the experiment. Software was loaded onto a Windows laptop with the Tobii Pro angled to face eye level of the participant.

The puzzle game was run on a 13-inch XPS laptop (Dell Technologies) with a Tobii Pro X3-120 (Tobii AB) eye-tracking bar attached below the screen facing upward at approximately the eye level of the participant (Figure 3). Both eyes were recorded in the eye-tracking process. Eye data were recorded at 120 Hz (maximum frequency) and a latency of <11 ms. This served as a corrective set of data for the Tobii Pro. The Tobii Pro software kit used by the Unity plugin would use these data to automatically correct the output data to an advertised accuracy of 0.4° at a rate of 120 Hz, precision 0.24° [48].

Participants were informed of the procedures in the user study, and written consent was obtained. This was followed by a cognitive assessment, where the MoCA, TMT-A, TMT-B, SnMT, and IADL were administered in paper-pencil format. For the puzzle-game session, participants were first instructed on how to play the SMT. Participants were told that there was only one target pattern to match for each board and were shown how to use the hint button.

The eye tracker was then calibrated, during which participants sat upright in front of the screen and a series of red dots appeared in each corner of the screen. After eye tracker calibration, a training block with 3 trials of incremental difficulty was administered (width, height, tiles = 4,4,4; 5,5,5; 6,6,6). In the test block, participants completed the 12 SMT difficulty levels. Players were instructed to complete each puzzle subset to advance to the next puzzle and that they could complete the puzzles at their own pace.

During their play through, eye tracking, mouse data, and puzzle-specific information was automatically recorded by the puzzle software. A summary file was automatically recorded for each participant, which included mouse data at 120 Hz, eye gaze data at 120 Hz, time taken to solve each puzzle, and which puzzle sets were chosen from a set of predetermined puzzles. All data were stored locally on the computer. On completion of the puzzle-game task, perception [45] and usability [46] of the game was collected in paper-pencil format.

### Data Analysis

As outcome variables, we calculated saccade distance, saccade duration, number of fixations (average, on target, and on distractor patterns), number of distractors, time for game completion, and visual and effective search time. The raw data were analyzed with Python 3.7 [49] and common libraries like pandas [50] and numPy [51]. Using the timestamps, we matched the content of the different files containing mouse, eye-tracking, and puzzle board data. The mouse data contains all the mouse movements and a flag indicating whether the mouse was clicked or not. The eye-tracking data files (*.xml) provide coordinates of the left and right eye on the screen, computed by the eye-tracking software. For the puzzle data, a summary file with entries for each played move on the SMT was stored. Each move entry in this summary file included the trial number, height, width of the puzzle board, number of unique tile types, move number, time to make the move, false or correct move, and hint use. Time-based game performance metrics were calculated as described in SMT publication [11].

Constraints for selecting fixations from the eye-tracking data:

- Fixation duration threshold had to be at least 100 ms, allowing a balance between theoretical maximum and minimum [52-56]
- Fixations with at least 5 data points available were considered for further analysis
- Points closer than 64 pixels to the previous point in the fixation were considered to segregate the region of interest on the board
- Fixations were discarded if the mean (average) of the left eye’s coordinates deviated more than 100 pixels from the mean of the right eye’s coordinates, setting a threshold from the centroid of fixation [57]
- Fixations were discarded if the standard deviation of the mean of all points was greater than 100 pixels following the position-variance threshold [28]

**Figure 4:**
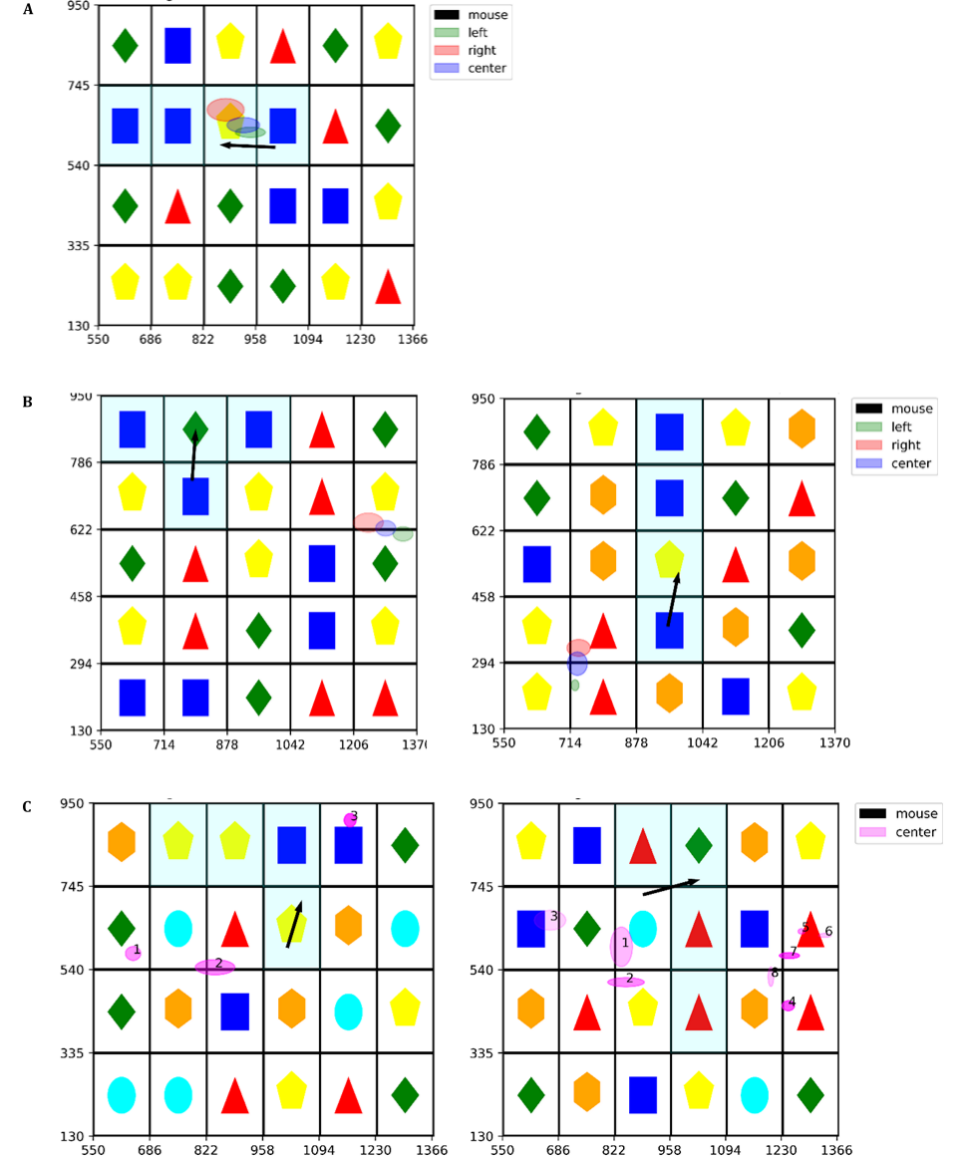
(A) Fixation of subject (participant ID 3, 66 years, male) while finding the correct match (target). The mouse drag (black) duration was 498 ms. (B) Single fixation on distractor patterns. Left: participant ID 10 (73 years, male, mouse drag duration 674 ms); right: participant ID 7 (70 years, female, mouse drag duration 763 ms). (C) Examples of all fixations on board until target pattern is found. Left: participant ID 3 (66 years, male, mouse drag duration 418 ms); right: participant ID 11 (71 years, female, mouse drag duration 744 ms). X and y axes show pixel values on screen. Black arrow shows movement and direction of clicked mouse. Target pattern (region of interest) is highlighted using transparent turquoise color. Center points of ellipses are the mean of the included fixation data points. Height and width of ellipses are derived from standard deviation in y and x direction of the included points, respectively. Duration of fixation is displayed with alpha value of the ellipses (the more transparent, the shorter the fixation).

For the saccades, we calculated the saccade duration as the difference between the start-timestamp of the current fixation and the end-timestamp of the last fixation. The saccade distance was derived from the Euclidean distance of the center point of the last fixation and the current fixation. The visual search time is calculated as the time from the board initiation until the first fixation on the target (for an example, please see Figure 4 in the results section). Effective search time corresponds to the mouse movement time. Game completion time is the total search time across all boards.

### Data Exclusion

Eye-tracking data of one participant (ID 9) were excluded due to insufficient data quality for analysis. Four additional participants (ID 2, 5, 12, and 13) were excluded due to the limited number of recorded fixations. The reason for this unusable data for our purpose might be some measurement errors or bad calibration of the eye-tracking system.

### Statistical Analysis

R-Studio version 1.1.463 (R Foundation for Statistical Computing) [58] was used for the statistical analysis. For the 8 participants with a sufficient amount of fixations, Spearman correlations were calculated, as this method doesn’t assume normally distributed data and is more robust toward outliers [59]. To help identify any variance accounted for by age and cognition, we performed additional partial correlations between the eye movement metrics and age as well as cognition. Effect sizes were estimated using Cramer *V* [60]. The signiﬁcance level was set at *P*<.05, as these were exploratory analyses we did not correct for repeated testing.

## Results

The eye-tracking bar seemed to provide accurate eye-tracking data with limitations discussed further on. The eye-tracking analysis included data from 8 subjects with sufficient number of fixations.

### Game Performance and Eye Movement Metrics

In total, 672 fixations were recorded for the complete dataset of the 8 subjects included in the analysis. Eye-tracking data analysis detected 99 fixations on target patterns and 525 on distractor patterns. Eye movement metrics and game performance scores for the 8 subjects included in the analysis are displayed in Table 2. There may be more than one fixation on targets because each target gem is counted as one target. So, if the fixation is on the edge of 2 target tiles it counts as 2 fixations on targets. Also, there might be multiple subsequent fixations on targets before the mouse was clicked (eg, ID 10 in Table 2).

Correlation analysis showed a tendency toward significance for game completion time with average fixations (*r_s_*=0.66, *P*=.08), fixations on distractors (*r_s_*=0.68, *P*=.06), and fixations on targets (*r_s_*=0.69, *P*=.06).

**Table 2:** Eye movement metrics and game performance of participants (n=8).

Subject	Visual search time (sec), mean (SD)	Effective search time (sec), mean (SD)	Fixations, mean (SD)	Fixations on distractors, mean (SD)	Fixations on targets, mean (SD)	Saccade duration (sec), mean (SD)	Saccade distance (sec), mean (SD)	Game completion time (sec)
1	—^a^	24.34 (17.37)	15.00 (23.39)	12.33 (18.77)	0 (0)	4.74 (5.51)	258.92 (168.92)	1290.29
3	16.61 (7.21)	40.73 (24.63)	2.40 (1.14)	2.00 (1.00)	0.40 (0.55)	12.62 (12.94)	259.72 (119.93)	1306.78
4	2.48 (0)	21.07 (15.87)	5.83 (6.49)	4.50 (6.83)	0.67 (1.63)	8.41 (5.70)	176.57 (82.54)	1495.21
6	1.59 (0)	5.25 (1.54)	1.00 (0)	1.00 (0)	0.33 (0.58)	5.53 (4.82)	125.25 (5.02)	430.72
7	8.84 (7.97)	19.02 (10.50)	7.20 (7.81)	5.53 (6.93)	0.80 (1.82)	3.81 (4.04)	136.10 (88.40)	1387.22
8	—	15.42 (0)	1.00 (0)	1.00 (0)	0 (0)	—	—	844.75
10	6.65 (4.98)	39.90 (42.71)	22.62 (40.62)	17.92 (35.26)	3.77 (7.70)	4.37 (5.73)	204.84 (141.76)	2180.39
11	14.48 (0)	17.79 (11.62)	5.33 (6.08)	4.56 (5.43)	0.33 (1.00)	3.08 (6.67)	268.10 (178.85)	1555.50

^a^Not available.

Figure 4A shows a single fixation (left, right, and center of left and right eye) of a subject after finding a match and just before dragging the mouse, while Figure 4B shows a single fixation on a distractor pattern. Figure 4C shows multiple fixations and fixation duration on a single board of 2 subjects until the target is found (left, right, and center of left and right eye). Duration of the fixation and number of fixations until the person finds the target match indicate the complexity of the tasks and the inhibition [61] involved in decision making.

### Set Size and Eye Movement Metrics

Eye movement metrics for the different set sizes are shown in Table 3. For a constant board height, visual search time increases with increasing width. Correlations between set size and visual search time (*r_s_*=0.18, *P*=.70), effective search time (*r_s_*=0.35, *P*=.39), number of fixations (*r_s_*=0.01, *P*=.98), fixations on distractors (*r_s_*=–0.15, *P*=.73), fixations on targets (*r_s_*=–0.42, *P*=.31), saccade distance (*r_s_*=–0.05, *P*=.91), and saccade duration (*r_s_*=0.24, *P*=.56) were not significant.

**Table 3:** Eye movement metrics for different board sizes.

Set size	Visual search time (sec), mean (SD)	Effective search time (sec), mean (SD)	Fixations on targets, mean (SD)	Fixations, mean (SD)	Fixations on distractors, mean (SD)	Saccade duration (sec), mean (SD)	Saccade distance (sec), mean (SD)
4×4	—^a^	—	—	—	—	—	—
4×5	7.58 (3.33)	10.13 (4.20)	0.80 (0.84)	3.40 (2.51)	2.20 (2.39)	5.10 (1.90)	220.40 (105.94)
4×6	8.79 (10.09)	26.66 (24.92)	1.62 (3.07)	5.38 (5.88)	4.50 (5.24)	4.14 (4.58)	197.43 (112.32)
5×4	3.39 (0)	18.30 (13.49)	2.33 (4.04)	14.33 (14.98)	13.33 (13.32)	2.86 (2.81)	171.73 (105.29)
5×5	6.39 (0)	16.43 (15.70)	0.14 (0.38)	3.57 (6.37)	3.29 (6.52)	4.20 (4.68)	301.03 (186.79)
5×6	—	29.36 (9.18)	0	4.00 (2.28)	1.17 (0.75)	6.99 (7.57)	195.54 (124.30)
6×4	8.77 (3.88	40.44 (53.05)	4.00 (10.15)	22.71 (54.37)	19.29 (46.62)	6.90 (8.28)	164.11 (97.49)
6×5	13.28) (10.25)	26.59 (32.77)	1.14 (1.68)	13.86 (22.00)	12.14 (18.75)	2.75 (4.84)	136.39 (93.59)
6×6	5.41 (5.32)	26.6x (12.31)	0.83 (2.59)	11.5x (12.03)	8.17 (9.42)	5.99 (8.10)	211.20 (144.48)

^a^Not available.

### Correlation of Eye Movement Metrics and Neuropsychological Assessment Scores

**Figure 5:**
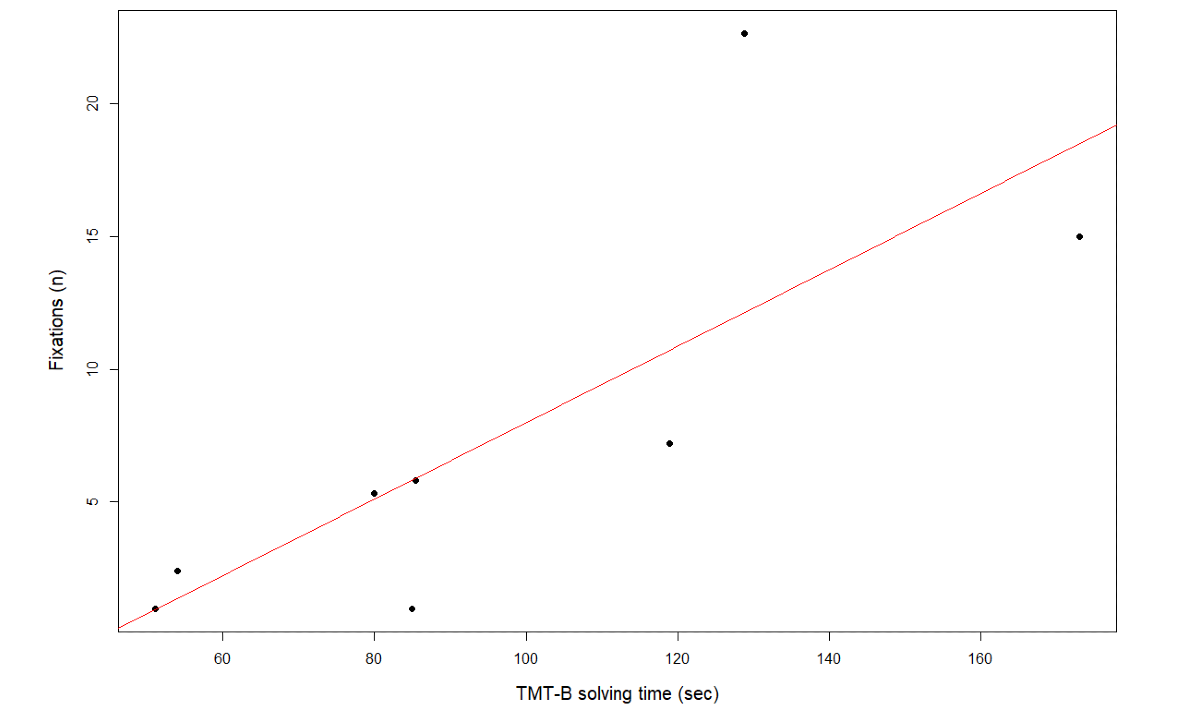
Correlation between average number of fixations and Trail Making Test B solving time.

Results of the correlation analyses can be seen in Multimedia Appendix 1. Executive functions measured by TMT-B solving time correlated significantly with number of fixations on distractors (*r_s_*=0.83, *P*=.01, Cramer *V*=0.2) and average number of fixations, (*r_s_*=0.87, *P*=.005, Cramer *V*=0.2, Figure 5), indicating a strong effect [60]. The cognitive subdomain MoCA recall correlated significantly with effective search time (*r_s_=–0.85, P*=.007), while MoCA attention associated significantly with saccade duration (*r_s_*=0.79, *P*=.03).

### Age as a Covariate

When controlled for age, executive functions measured by TMT-B solving time correlated significantly with number of fixations on targets (*r_s_*=0.97, *P*=.03), average number of fixations, (*r_s_*=1.0, *P*<.001), and effective search time (*r_s_*=0.96, *P*=.04). In addition, functional health measured by IADL associated significantly with average number of fixations (*r_p_*=–0.97, *P*=.03), fixations on distractors (*r_p_*=–0.97, *P*=.03), and fixations on distractors (*r_p_*=–0.99, *P*=.008). However, IADL measurements showed no significant correlation with effective search time (*r_p_*=–0.939, *P*=.06).

## Discussion

The SMT puzzle game has the potential to target the following cognitive domains: learning and memory (working memory), attention (visual search), executive functions (inhibition and flexibility), and perceptual-motor function (visuospatial ability), both as a training and diagnostic tool. The analyzed game metrics after game play (ie, game completion time) have been validated against some of the cognitive domains in a previous study [11]. In this study, we explored the feasibility of supplementing puzzle games with the method of eye-tracking. Eye tracking offers the possibility to observe eye movements in real-time providing additional information about cognitive processes that may go undetected with game testing alone.

### Principal Findings

We focused on associations between eye-tracking measures and global cognition, attention executive functions, and game performance measures. The results of this preliminary study indicate an effect of age on number of fixations and found significant correlations between executive functions (TMT-B solving time) and number of fixations, as well as cognitive subdomains recall and attention with effective search time and saccade duration respectively.

### Comparison With Prior Work

Eye-tracking devices vary in how eye movements are measured, sampling rate of eye position, accuracy, allowance of head movements, and ease of use. The eye-tracking bar used in this study recorded gaze data at a rate of 120 Hz, which falls within research practices of using 25 Hz to 250 Hz to investigate higher-level cognition [62] allowing free movement of head. While these data are still useful, increasing the fidelity of this data collection may also yield a finer detail of gaze data. The sampling rate of 120 Hz may provide sufficient data points for tracking fixations and barely enough for fixation duration but may fall short for researching saccades [62]. In our sample, the quality of eye-tracking data for some participants was low (ie, few data points were being recorded and were nonsuitable for analysis). Previous research has shown that participant calibrated eye-tracking systems provide higher data quality than operator or automatically calibrated systems. In addition, corrective glasses are known to impair eye-tracking data precision [63]. Both factors could have had a negative effect on data quality in our sample, as many participants wore glasses and we used automatic calibration for eye tracking.

Visual fixation in continuous visual tasks varies in duration (from less than 100 ms to several seconds) [54]. A fixation is an interplay of the visual, oculomotor, and cognitive systems, and hence fixation duration can result from components of inhibition, attention, and cognitive control. The proportion or count of very short fixations can infer the inhibitory influence of the cognitive system [61]. The constraint of 100 ms in our fixation analysis, although on the lower end, gives us the possibility to also include short fixations on distractors wherein the contextual information processed is less. Our analysis resulted in a total of 672 fixations, which led to a low value per puzzle board, considering the puzzle board size and number of participants. Moreover, the total number of fixations was larger than the sum of number of fixations on targets and distractors. This can be explained by fixations on other tile types as well as fixations on edges between targets and distractors that were counted as two fixations. It’s questionable if fixations are the appropriate metric for such analysis or if the constraints selected or the region of interest were correct. Our analysis also looks at the saccades in between the fixations; however, saccades are less driven by the cognitive content of the visual stimuli.

The setup used in this study required a mouse as the input device for swapping tiles. To end one board in the puzzle game, a matching target had to be found with a mouse drag. Mouse movement time (ie, effective search time) and visual search time both contributed to game completion time. Lack of association of fixations with game completion time can be attributed to the small set of viable fixation data as well as the motor component involved from the mouse movement.

The difficulty levels in this study were presented in a random order to avoid learning effects. Difficulty level is a number coded for a unique combination of set size and number of types of tiles. Set size effects on search time are well documented. Previous publications from this project [11] report that search time increases with set size, as finding a target pattern in a larger board requires more search and scan time. The previous study [11] also reports that finding a target pattern is less difficult when there are more different types of tiles on the puzzle board. In this study, the average number of fixations, fixations on distractors, and fixations on targets decreases with increasing set size following the trend of set size effect, while visual search time does not follow the same trend.

In the TMT-B task, letters and numbers must be connected alternating in increasing order. Our results revealed significant associations between fixations and measures of divided attention (TMT-B) as well as age. This agrees with findings from earlier studies that reported relationships between TMT-B solving time and visual search measures, attention [64,65], and executive functions (eg, cognitive flexibility [42] and working memory [66]). Visual search and attention are fundamental abilities for solving the puzzle tasks in this study. The association of TMT-B with fixations after controlling for age follows previous research that performance in the TMT task is affected by increasing age [67], and performance on a TMM3 puzzle game is related to measures of selective and divided attention in older adults [68]. The correlations in our data indicate that eye tracking can provide data about cognitive functions, and eye-tracking measures are a possible way to depict these processes.

In the past, several visual exploration studies have investigated eye movements in different populations; however, limited studies are reported for puzzle games. One study with children related eye movements to performance in a puzzle game. They assumed that better performance in the game was associated with different patterns of eye movement than those of lower performing participants. While the results did not allow a clear conclusion regarding fixation duration, it seems lower performers showed higher fixation density in most cases. Fixation intensity also decreased when performance in new levels increased [69]. To our knowledge, there exist no studies performing eye tracking during a puzzle game in older adults. In our study, participants with longer SMT-solving times (ie, low performance in this test) performed more fixations on distractors. This could be a sign of more inefficient visual search behavior, which could be related to a decline in executive functions.

Lack of a relationship between general cognition and eye-tracking measures might be explained by the high MoCA scores of our sample. They ranged from 26 to 30 points, which confirms the inclusion of cognitively nonimpaired participants in the study but is probably too small to find correlations. As mentioned in the introduction, other studies with cognitively impaired patients did find a relationship between cognition and eye movements [37,38,69], moderated by the amount of cognitive impairment [10]. Therefore, we assume that a similar study in patients with a diagnosis of dementia might show a relationship between MoCA scores and eye movements.

### Limitations

One limitation was that participants wearing glasses had calibration difficulties with the eye-tracking bar, reducing the available participant data. Another limitation was that even after successful calibration, we could not record any fixation on a target in some cases, leading to more sparse data in the visual search time (cells marked as not available in Table 2). The main limitation was the small sample size, which posed a risk of false significant results. Although our participants were healthy older adults with little or no prior game experience, it remains unclear whether our findings can be generalized to a larger population and cognitively impaired persons. But the fact that most significant correlations are related to TMT-B solving time indicates that there exists some relation between executive functions and eye-tracking measures.

### Outlook

#### Clinical

Eye movements are known to be affected in neurodegenerative diseases. In Huntington disease, abnormal eye movements are one of the earliest manifestation of the disease [70]. Previous research has shown that patients diagnosed with dementia or mild cognitive impairment show more and longer fixations on distractor items than healthy participants [38]. Moreover, the visual search strategy of patients with dementia focuses more strongly on areas in the periphery, while healthy older participants focus more strongly on the center of the visual field [71]. The repetition of this study in samples affected by pathological aging could confirm these findings. The combination of eye tracking and a puzzle game provides the option of a cognitive assessment in a game-like fashion, which could be less stressful for the patient. Additionally, this setting is probably not affected by level of education and literacy, which is the case in assessments like the MoCA [72].

#### Technical

For further research and applying eye tracking during cognitive assessments, a better approach to obtaining eye-tracking data is likely to be found using eye-tracking glasses rather than an eye-tracking bar. There are eye-tracking glasses available that can be used with participants using glasses. This will solve the issue of blocking the eye-tracking bar with participants performing the task on tablets. By placing ArUco markers [73] in the corners of the screen of the tablet app, it would be possible to map recorded eye-tracking data onto the screenspace of the tablet and obtain unobstructed data while also retaining motor function data from the touch display.

#### Data Analysis

From the data collected, it would also be possible to recreate real-time playback of the session with fixations of the moment showing in overlay. This could provide a valuable visual of the participant’s attention to distractors and solutions and avoid any scoring errors in case of doubts.

### Conclusions

Eye tracking is a feasible way to collect an extra subset of relevant data. Stationary eye tracking can be used for the recording of visual search in a study of cognitive puzzle games presented on laptops or desktops. However, other eye-tracking devices are preferable for use with tablets, as touch interactions don’t block the eye tracker and are more likely to calibrate easier for participants with glasses. The approach shown in this study takes advantage of the eye movement metrics (fixations and saccades) to assess cognitive abilities in a game setup. Fixations show potential as adjunct digital markers for executive functions in puzzle game tasks. These supplementary data can provide additional information to develop cognitive markers. If rigorously tested and evaluated in larger cohorts, it will enhance the specificity and sensitivity of the puzzle game as a diagnostic tool. Further, this study demonstrated that saccades and fixations can be susceptible to aging. Future research should strive to include more age groups and also include cognitively impaired participants to allow generalizability.

## Appendix

Multimedia Appendix 1 Correlations between subject characteristics and eye movement metrics.

## Abbreviations

AD: dementia due to Alzheimer disease
Lawton-IADL: Lawton Instrumental Activities of Daily Living
MoCA: Montreal Cognitive Assessment
TMM3: tile-matching match-three
SMT: Search and Match Task
SnMT: Snellgrove Maze Test
TMT: Trail Making Test
