# Supplementary material for: Application of Eye Tracking in Puzzle Games for Adjunct Cognitive Markers: Pilot Observational Study in Older Adults
Source: JMIR Serious Games. 2021 Mar 22;9(1):e24151. doi: 10.2196/24151 (PMC8078028; doi:10.2196/24151)
Supplement: Multimedia Appendix 1 [file games_v9i1e24151_app1.docx]

Multimedia Appendix 1. Correlations between subject characteristics and eye movement metrics.

| Predictors | TMT- A | TMT-B | SnMT | MoCA | Age |
| --- | --- | --- | --- | --- | --- |
| Fixations (n) | *r_s_* = 0.49 *P* = .22 | *r_s_* = 0.87 *P* = .005**^a^** | *r_s_* = - 0.59 *P* = .16 | *r_s_* = - 0.40 *P* = .33 | *r_s_* = 0.76 *P* = .03**^a^**, V = 0.2 |
| Fixations on Distractors (n) | *r_s_* = 0.54 *P* = .17 | *r_s_* = 0.83 *P* = .01**^a^** | *r_s_* = - 0.56 *P* = .19 | *r_s_* = - 0.28 *P* = .50 | *r_s_* = 0.69. *P* = .06 |
| Fixations on Targets (n) | *r_s_* = - 0.17 *P* = .69 | *r_s_* = 0.19 *P* = .65 | *r_s_* = 0.02 *P* = .97 | *r_s_* = 0.13 *P* = .76 | *r_s_* = 0.27 *P* = .51 |
| Visual Search Time (sec) | *r_s_* = 0.20 *P* = 0.71 | *r_s_* = 0.03 *P* = 1**^a^** | *r_s_* = - 0.70 *P* = .23 | *r_s_* = - 0.32 *P* = .54 | *r_s_* = -0.09 *P* = .92 |
| Effective Search Time (sec) | *r_s_* = 0.14 *P* = .75 | *r_s_* = 0.43 *P* = .30 | *r_s_* = - 0.46 *P* = .30 | *r_s_* = - 0.58 *P* = .13 | *r_s_* = 0.40 *P* = .33 |
| Saccade Duration (sec) | *r_s_* = -.50 *P* = .25 | *r_s_* = - 0.36 *P* = .43 | *r_s_* = - 0.03 *P* =.96 | *r_s_* = - 0.36 *P* = .42 | *r_s_* = -0.09 *P* = .85 |
| Saccade distance (sec) | *r_s_* = 0.61 *P* = .15 | *r_s_* = 0.11 *P* = .82 | *r_s_* = 0.14 *P* = .79 | *r_s_* = - 0.40 *P* = .37 | *r_s_* =0.20 *P* = .67 |
| Game completion time (sec) | *r_s_* = 0.55 *P* = .17 | *r_s_* = 0.38 *P* = .36 | *r_s_* = 0.18 *P* = .71 | *r_s_* = - 0.18 *P* = .66 | *r_s_* = 0.66 *P* = .08 |

r_s_ = Spearman correlation coefficient, ^a^Significant at P < .05, V = Cramer’s V. MoCA = Montreal Cognitive Assessment, TMT= Trail Making Test, SnMT = Snellgrove Maze Test.
